# Fluoropolymer Functionalization of Organ-on-Chip Platform Increases Detection Sensitivity for Cannabinoids

**DOI:** 10.3390/bios13080779

**Published:** 2023-08-01

**Authors:** Ziqiu Tong, Lars Esser, Peter Galettis, David Rudd, Christopher D. Easton, Azadeh Nilghaz, Bo Peng, Douer Zhu, Helmut Thissen, Jennifer H. Martin, Nicolas H. Voelcker

**Affiliations:** 1 Drug Delivery, Disposition and Dynamics, Monash Institute of Pharmaceutical Sciences, Monash University, Parkville, VIC 3052, Australia; tommy.tong@monash.edu (Z.T.); david.rudd@monash.edu (D.R.); a.nilghaz@monash.edu (A.N.); iambpeng@nwpu.edu.cn (B.P.); douer.zhu@monash.edu (D.Z.); 2 Commonwealth Scientific and Industrial Research Organisation (CSIRO), Clayton, VIC 3168, Australia; lars.esser@csiro.au (L.E.); chris.easton@csiro.au (C.D.E.); helmut.thissen@csiro.au (H.T.); 3 Centre for Drug Repurposing & Medicines Research, School of Medicine and Public Health, Faculty of Health, Medicine & Wellbeing, The University of Newcastle, Callaghan, NSW 2308, Australia; peter.galettis@newcastle.edu.au; 4 Centre Hunter Medical Research Institute, New Lambton Heights, NSW 2305, Australia; 5 Institute for Frontier Materials, Deakin University, Waurn Pounds, VIC 3216, Australia; 6 Xi’an Institute of Biomedical Materials & Engineering, Northwestern Polytechnical University, Xi’an 710072, China; 7 Melbourne Centre for Nanofabrication, Victorian Node of the Australian National Fabrication Facility, Clayton, VIC 3168, Australia; 8 Materials Science and Engineering, Monash University, Clayton, VIC 3168, Australia

**Keywords:** cannabinoids, cannabidiol, microfluidics, organ-on-chip systems, detection sensitivity, tetrafluoroethylene

## Abstract

Microfluidic technology is applied across various research areas including organ-on-chip (OOC) systems. The main material used for microfluidics is polydimethylsiloxane (PDMS), a silicone elastomer material that is biocompatible, transparent, and easy to use for OOC systems with well-defined microstructures. However, PDMS-based OOC systems can absorb hydrophobic and small molecules, making it difficult and erroneous to make quantitative analytical assessments for such compounds. In this paper, we explore the use of a synthetic fluoropolymer, poly(4,5-difluoro-2,2-bis(trifluoromethyl)-1,3-dioxole-*co*-tetrafluoroethylene) (Teflon™ AF 2400), with excellent “non-stick” properties to functionalize OOC systems. Cannabinoids, including cannabidiol (CBD), are classes of hydrophobic compounds with a great potential for the treatment of anxiety, depression, pain, and cancer. By using CBD as a testing compound, we examined and systematically quantified CBD absorption into PDMS by means of an LC-MS/MS analysis. In comparison to the unmodified PDMS microchannels, an increase of approximately 30× in the CBD signal was detected with the fluoropolymer surface modification after 3 h of static incubation. Under perfusion conditions, we observed an increase of nearly 15× in the CBD signals from the surface-modified microchannels than from the unmodified microchannels. Furthermore, we also demonstrated that fluoropolymer-modified microchannels are compatible for culturing hCMEC/D3 endothelial cells and for CBD perfusion experiments.

## 1. Introduction

Over the past decade, we have seen explosive progress in the field of organ-on-chip (OOC) technology [1]. OOC systems aim to replicate the characteristics of human or animal tissue counterparts to measure a variety of key physiological functions of native organs. This is achieved by controlling the microphysiological environments (e.g., a microfluidic construct) that the cells are cultured in. Microfluidic constructs are built using various biocompatible materials. Some examples of vital human organ counterparts that have been extensively explored in OOC systems include the heart [2], liver [3], skin [4], kidney [5], lung [6], intestine [7], and biological barriers [8], among others. Interconnecting organ mimics has also proven to be advantageous and can provide more accurate results than testing on an individual module unit of an OOC system [9]. For example, an interconnected multi-OOC system can be beneficial towards the assessment of how the human body absorbs, distributes, metabolizes, and eliminates (ADME) drugs [10]. Sung and colleagues reported the use of a multi-OOC system consisting of a fluidic network of four chamber compartments mimicking the circulatory system (lung, liver, fat tissue, and other tissue) for testing the metabolism-dependent toxicity of several drugs, such as Tegafur [11]. Furthermore, OOC technology enables the development of different models of human diseases, such as neurological diseases [12]. These OOC systems can also be integrated as biosensors or diagnostic tools for disease management, such as long COVID management using a SARS-CoV-2 on-chip [13]. Not surprisingly, due to their potential transformative capabilities for the field of drug discovery, OOC technology has already attracted significant commercial interest, and some startup companies have recently emerged, such as Emulate™ and Hesperos™ [14].

PDMS has been a popular material for OOC devices since the beginning of the field of OOC technology [15]. There are several important key features that PDMS possesses that still make it a material of choice for OOC devices today. The advantages of using PDMS include a relatively low cost; high elasticity allowing for excellent chip manipulation; optical transparency, which makes it suitable for cell imaging; high gas permeability; and biocompatibility for long-term cell culture [16]. In terms of device fabrication, PDMS allows for the fabrication of a flexible membrane and microchannels, and conforms to various topographies with high resolutions [15]. Despite PDMS being one of the most popular materials for constructing OOC devices, there are some inherent drawbacks associated with PDMS that need to be overcome. For example, if the PDMS monomer is not completely cured during the fabrication process, the leaching of uncured oligomers from the polymer network into microchannel media can be toxic for cell culture [17]. Another important drawback is the hydrophobic nature of the PDMS material, which can lead to a significant absorption of small hydrophobic molecules, including components from cell culture media and from the test compounds being assessed in cell–drug interaction studies in OOC systems [17]. As a result, utilizing PDMS-based OOC devices can lead to incorrect ADME values being derived.

Therefore, various PDMS surface modification strategies have been recently explored to improve its utility and to expand its use in OOC applications [18,19,20]. For example, using oxygen plasma treatment, the PDMS surface can be temporarily converted from hydrophobic to hydrophilic. Tan et al. demonstrated the use of a scanning radical microjet approach with an oxygen plasma to maintain the PDMS device’s hydrophilicity for several weeks [21]. Surface modification via physisorption can be experimentally simple and quick. For example, the physical adsorption of a triblock copolymer, polyethyleneoxy (POE)-polyoxypropylene (POP)-POE, Pluronic F108, was achieved on a PDMS surface [22]. The hydrophobic POP part of the F108 was attached to the hydrophobic PDMS surface, whereas the hydrophilic POE part was exposed to the solution [22]. However, the weak interactions between the surface and the surface-modifying agent can result in thermal and mechanical instabilities. On the other hand, chemical modifications, such as functionalization with silanes, can offer a long-term stability and achieve better surface robustness. For example, Chuah et al. reported a method to modify a PDMS surface with (3-aminopropyl)triethoxy silane (APTES) and glutaraldehyde crosslinking to reduce PDMS hydrophobicity and to increase protein immobilization [23].

Other alternative materials, such as alginate, thermoplastics, ceramics, and resin, have also been actively tested for suitability in OOC applications [24]. In particular, the use of microfluidic chips fabricated entirely from Teflon was reported by Ren et al., presenting no absorption of small molecules and no leaching of residue molecules [25]. However, the process of fabricating such Teflon chip involves the use of a hot embossing technique requiring a high temperature and cannot achieve the same high resolution as PDMS material. To sum, the above-mentioned surface functionalization techniques usually require several complex modification steps, and other alternative materials do not have the same highly desirable features as PDMS. Hence, a facile surface modification of PDMS, which can yield a high degree of hydrophobic compound recovery and at the same time provides “inertness” in the coating, is highly desirable.

Cannabinoids, such as cannabidiol (CBD) and Δ9-tetrahydrocannabinol (THC), are highly hydrophobic compounds and can readily distribute into adipose tissues and other active surfaces [26]. Cannabis and its derivatives are increasingly being investigated in preclinical models and clinical studies for the treatment of a range of diseases [26]. For example, cannabinoids have been trialed in cancer patients and have shown some promising preclinical data, suggesting their potential as anticancer agents [26]. Cannabinoids have also been explored in the areas of pediatric seizures, neuropathic pain, and multiple sclerosis [27]. CBD, which is less psychoactive than THC, and other cannabinoids have been commercialized as analgesic, anti-inflammatory, anti-wrinkle, and moisturizing agents [28]. The use of CBD for treating skin diseases and inducing hair regrowth has also been explored [29].

The common practice of screening the initial efficacy of CBD and other cannabinoids against different disease types still heavily relies on the use of either animal-based in vivo models [30,31,32] or traditional well plate cell culture methods [33,34]. Due to the inherent variation between different species and the simplicity of 2D cell culture, traditional methods for drug testing have been deemed as inefficient [9]. “Humanized” OOC systems can become the next gold standard for testing drug efficacies. Despite the recent popularity of using cannabinoids in medical settings, to the best of our knowledge, we have yet to see any reports on the testing of cannabinoids in OOC systems. The absorption of hydrophobic compounds, such as cannabinoids, in PDMS-based OOCs is a critical issue that needs to be addressed. Additionally, methods to improve the suitability of the microfluidic system in delivering CBD are also likely to be beneficial to numerous classes of lipophilic drugs.

In this paper, we demonstrate the use of a simple one-step functionalization to passivate PDMS surfaces with a thin coating of the fluoropolymer Teflon AF 2400. CBD absorptions on flat PDMS surfaces and in microfluidic channels functionalized with and without the fluoropolymer were compared. The azo coupling derivatization method was applied to the post-collection of CBD to increase the CBD detection limit via LC-MS/MS. Finally, human endothelial cells, hCMEC/D3, were cultured in PDMS microchannels to test the biocompatibility of surfaces coated with Teflon AF 2400. In combination with the hCMEC/D3 cell monolayers, the microchannels coated with the fluoropolymer produced a detection sensitivity towards CBD that was eight times higher in comparison to the unmodified microchannels. This straightforward, one-step PDMS surface modification in microfluidic channels can be useful for OOC applications in order to achieve an improved hydrophobic compound recovery.

## 2. Materials and Methods

### 2.1. Chemicals

Ammonium formate, ammonium acetate, Fast Red RC Salt, formic acid, methanol hypergrade for LC-MS, hexamethyldisilazane, and Poly [4,5-difluoro-2,2-bis(trifluoromethyl)-1,3-dioxole-co-tetrafluoroethylene] (Teflon™ AF 2400) were purchased from Sigma-Aldrich (Saint Louis, MO, USA). Fluorinert FC-72 was purchased from SynQuest Laboratories (Alachua, FL, USA).

### 2.2. PDMS-Based Microfluidic Channel Fabrication

The master mold for PDMS was fabricated via the standard soft photolithography technique as previously described [35]. Initially, a silicon wafer was cleaned using acetone and isopropanol and prebaked at 200 °C for 2 min. To achieve a height of 125 µm, a photoresist SU-8 50 was spun onto the wafer using a Karl Suss Delta 80 spin coater (Suss MicroTec, Garching, Germany) at 2000 rpm for 30 s. The wafer was subsequently transferred to a hot plate and baked at 65 °C for 4 min, followed by baking at 95 °C for 15 min. The silicon wafer coated with SU-8 50 was then exposed to 350 mJ/cm^2^ of UV using the EVG 620 mask aligner. The wafer was further baked at 65 °C for 2 min and at 95 °C for 8 min. The pattern was developed using propylene glycol methyl ether acetate before undergoing hard baking at 65 °C, 95 °C, and 130 °C for 1, 3, and 10 min, respectively. Finally, the SU-8 patterned silicon wafer was treated with hexamethyldisilazane inside a vacuum desiccator at 130 °C for 30 min.

For the PDMS-based microfluidic device fabrication, a mixture of polydimethylsiloxane prepolymer (Dow Chemical, Midland, MI, USA) and curing agent at a 10:1 ratio was thoroughly mixed and then poured onto the SU-8 patterned silicon wafer and degassed under vacuum. To produce flat PDMS pieces, the mixture of PDMS prepolymer and catalyst was poured onto a Petri dish and degassed under vacuum. The PDMS was then cured in an oven at 80 °C for 4 h and cut out using a surgical knife before peeling off. The inlet and outlet ports were punched out using a 1.5 mm diameter Harris Uni-Core puncture (Ted Pella, Redding, CA, USA). The finished PDMS channels were then cleaned via sonication in ethanol before being irreversibly bonded to glass coverslips (Proscitech, G418, No1) using a 40 s oxygen plasma cleaner (Harrick Plasma, Ithaca, NY, USA) to create microfluidic devices. Finally, the devices were heat treated at 80 °C for 10 min to obtain permanent bonding. The flat PDMS pieces were cut directly from the Petri dish and cleaned via sonication in ethanol before use.

### 2.3. Coating Flat PDMS Surfaces and Microchannels with Teflon AF 2400

Teflon AF 2400 pellet was dissolved in Fluorinert FC-72 to obtain an initial stock concentration of 2% with sonication in an ultrasonic water bath (Elma S60, Singen, Germany) to help with the dissolution. The stock solution was then further diluted to 1% and 0.5% using Fluorinert FC-72. An amount of 20 µL of Teflon AF 2400 solutions at different concentrations (2%, 1%, 0.5%, and 0%) were pipetted onto flat PDMS pieces and incubated for 30 min in an oven at 80 °C. Similarly, 10 µL of Teflon AF 2400 solution (1%) was injected into microfluidic channels using a pipette and then placed in an oven for 30 min at 80 °C. The coated flat PDMS pieces and microfluidic channels were washed with Fluorinert FC-72 solvent and then dried again in an oven for another 15 min.

### 2.4. Water Contact Angle (WCA) Measurement

The WCA of PDMS surfaces used in this study were measured using an optical tensiometer (KSV CAM 200) at 25 °C. A ~3 μL drop of Milli-Q water was carefully placed on the uncoated PDMS surface and on the surface of PDMS coated with Teflon AF 2400. Images were captured, and WCA values were automatically calculated from the acquired images via the built-in software. All measurements were repeated at least three times, and the results were averaged.

### 2.5. Attenuated Total Reflection-Fourier Transform Infrared Spectroscopy (ATR-FTIR)

ATR-FTIR spectra of the uncoated PDMS surface and the PDMS surface coated with Teflon AF 2400 were obtained using a Thermo Scientific Nicolet 6700. A diamond crystal was run in ATR configuration with a 2 mm diamond tip and a deuterated triglycine sulphate detector. The spectra collected were averaged from 64 recorded scans with a resolution of 8 cm^−1^. Background spectra were blanked using air. The data were processed using OMNIC software.

### 2.6. X-ray Photoelectron Spectroscopy (XPS)

XPS analysis was conducted to confirm the surface chemistry of the fluoropolymer-modified PDMS. XPS was performed using an AXIS Nova spectrometer (Kratos Analytical Inc., Manchester, UK) with a monochromated Al Kα source at a power of 180 W (15 kV × 12 mA) and a hemispherical analyzer operating in the fixed analyzer transmission mode with the standard aperture (analysis area: 0.3 mm × 0.7 mm). The total pressure in the main chamber during analysis was typically between 10^−9^ and 10^−8^ mbar. Survey spectra were acquired at a pass energy of 160 eV. The samples were filled into the shallow wells of a custom-built sample holder and were analyzed at a nominal photoelectron emission angle of 0° with respect to the surface normal.

Data processing was performed using CasaXPS software version 2.3.15 (Casa Software Ltd., Teignmouth, UK). All present elements were identified from survey spectra. The atomic concentrations of the detected elements were calculated using integral peak intensities and the sensitivity factors supplied by the manufacturer. Binding energies were referenced to the C 1s peak at 285.0 eV (aliphatic hydrocarbon).

### 2.7. CBD Incubation on Flat PDMS and Inside Microfluidic Channels

CBD powder (Cerilliant, Round Rock, TX, USA) was dissolved in DMSO at 10 mg/mL as stock concentration and was further diluted in Milli-Q water to 1 µg/mL. An amount of 10 µL of CBD at 1 µg/mL was dispensed and incubated on the flat PDMS pieces coated with Teflon AF 2400 for 3 h inside a cell culture incubator (37 °C). In the case of testing the CBD absorption into the microfluidic channels, 10 µL of CBD at 1 µg/mL was manually injected into the microchannel and incubated for 3 h at 37 °C. The inlet and outlet of the microchannel were taped to prevent evaporation. CBD solution inside the microchannel was then collected for analysis by means of LC-MS/MS. In selected experiments, CBD solution was perfused into the microchannel coated with and without Teflon AF 2400 by using a syringe pump (Harvard Apparatus, Holliston, MA, USA). A disposable plastic syringe made from polypropylene (Terumo, Tokyo, Japan) and Tygon™ ND-100-80 tubing (Masterflex, Gelsenkirchen, Germany) was used for perfusion in selected experiments to test the adsorption of CBD onto plastic surfaces for 3 h perfusion. In most of the perfusion experiments presented in this paper, a glass syringe (Sanitex, SG Scientific, NSW, Australia) and polyetheretherketone (PEEK) polymer tubing (Restek, Centre County, PA, USA) were used instead. The eluent samples of 10 µL were collected and later subjected to derivatization and LC-MS/MS analysis.

### 2.8. CBD Derivatization and LC-MS/MS Analysis

The derivatization of CBD was carried out to increase the ionization efficiency of CBD and therefore enhance the sensitivity for detecting CBD. Fast Red RC was dissolved in ammonium acetate (5 mM) at a concentration of 100 µg/mL. The CBD samples collected (10 µL) from the flat PDMS and from inside of the microfluidic channels were mixed with Fast Red solution at a 1:1 ratio in a glass vial insert (Shimadzu, Kyoto, Japan) and incubated for 10 min, forming azo-coupling-derivatized CBD before injection into a Shimadzu triple quadrupole LC-MS/MS (Shimadzu 8050) for analysis. Note that the excess molar ratio of the derivatization agent was used to ensure the complete derivatization of the CBD samples.

The LC-MS/MS method was modified by Luo et al. [36]. Mobile phase A consisted of 5.0 mM ammonium formate in Milli-Q water and 0.05% formic acid. Mobile phase B consisted of LC-MS grade methanol with 0.05% formic acid. Chromatography was achieved on an Ascentis^®^ C18 HPLC column (Merck, Rahway, NJ, USA) with a 3 µm particle size (15 cm × 2.1 mm). The following gradient elution was employed: 0–1 min at 95% mobile phase B, 1–4.5 min at 100% mobile phase B. The sample injection volume was 2 µL, with a flow rate of 0.50 mL/min, and the column was kept at 40 °C. To eliminate carry-over, a needle rinse dip time of 30 s was used. The following MS conditions were applied: ESI source in positive ion mode, interface voltage at 2 kV, interface temperature at 300 °C, nebulizing gas (N_2_) flow at 2 L/min, DL temperature at 250 °C, heat block temperature at 400 °C, and drying gas (air) flow rate at 10 L/min. Multiple reaction monitoring (MRM) transitions for derivatized CBD were as follows: *m*/*z* 483.10 → 157 (collision energy −35.0 V), *m*/*z* 483.10 → 270.3 (collision energy −25.0 V). A total ion chromatogram (TIC) was created by summing these two main transitions, and the area under the curve of the TIC was used to quantify the amount of derivatized CBD. In a typical LC-MS analysis, one of the transitions would be used as the quantifier, and the other would be used as the qualifier. However, since our samples were clean enough (CBD directly dissolved in aqueous solution and not in a complex matrix), using the TIC as the quantifier could be justified, and yielded a more sensitive quantification.

### 2.9. hCMEC/D3 Cell Culture in Microchannels Coated with and without Teflon AF 2400

The immortalized human brain microvascular endothelial cell line (hCMEC/D3, Merck, NJ, USA) was cultured in growth basal medium-2 (EBM-2, Lonza, Basel, Switzerland) with supplemented growth factors as previously described [12]. hCMEC/D3 cells were cultured on tissue culture flasks coated with collagen-I (10 μg/mL, rat tail, Sigma-Aldrich). Once confluency was reached, hCMEC/D3 cells were dissociated from the flask using Accutase™ cell dissociation reagent (Thermo Fisher, Waltham, MA, USA) for subsequent maintenance and seeding into microchannels.

Microchannels coated with and without 1% Teflon AF 2400 were incubated with 1:10 diluted Matrigel (Sigma, Saint Louis, MO, USA) solution overnight. hCMEC/D3 cells were detached from the cell culture flask and resuspended in the culture medium at a concentration of 1 × 10^6^ cells/mL, and the cell suspension was injected into microfluidic channels. Cells were cultured inside an incubator (5% CO_2_ and 37 °C) for 2 days before being subjected to immunostaining or to CBD treatment under flow. A syringe pump equipped with a glass syringe and PEEK tubing was connected to the cell channel and perfused with CBD solution (1 μg/mL) at 2 μL/min for 3 h. Upon the completion of the experiments, the eluents were collected, and 10 μL of the collected CBD solutions were derivatized and analyzed by means of LC-MS/MS.

### 2.10. Cell Staining

hCMEC/D3 cells were cultured inside the microfluidic channels (modified with and without Teflon AF 2400 coating) for 2 days and then the cells were washed with PBS and fixed with 4% formaldehyde in PBS for 10 min. Cells were then rinsed with PBS three times, followed by permeabilization with Triton X-100 solution (0.1%) for 10 min. A staining solution consisting of 4′,6-diamidino-2-phenylindole (DAPI, 1 µg/mL) (Thermo Fisher, Waltham, MA, USA) and Phalloidin-TRITC (Phalloidin, 50 µg/mL, Sigma) was pipetted into the microfluidic channel and incubated for 1 h. The cells were then washed with PBS before being subjected to imaging analysis with an SP8 confocal microscope (Leica Microsystems, Wetzlar, Germany).

### 2.11. Data Analysis

All reported data were conducted in triplicate (N = 3) unless otherwise stated. Student’s *t*-test was used to compare statistical significance between each condition using Prism V9.5.1, and graphs were also generated using Prism V9.5.1.

## 3. Results and Discussion

**Figure 1:**
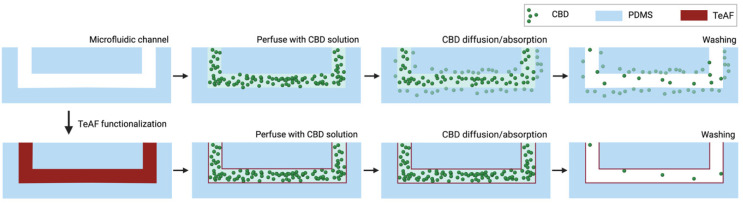
Schematic depiction of CBD diffusion and absorption into PDMS-based microfluidic chip system. When microfluidic channel is passivated with poly [4,5-difluoro-2,2-bis(trifluoromethyl)-1,3-dioxole-co-tetrafluoroethylene] (Teflon™ AF 2400), the absorption of CBD into PDMS is reduced. TeAF: Teflon™ AF 2400. Created using BioRender.com (accessed on 1 August 2022).

The use of PDMS as a constructing material for microfluidics, particularly for OOC application, has been instrumental; however, the absorption of small molecules (especially hydrophobic drug compounds) into PDMS has hampered the widespread use of PDMS [17]. We explored the use of the Teflon AF 2400 fluoropolymer to passivate the microfluidic channel surface to prevent small molecule binding (Figure 1). The fluoropolymer forms a thin Teflon-like layer on the PDMS surface after 30 min of incubation. CBD, which is a highly hydrophobic cannabinoid compound, was used to quantify the extent of hydrophobic compound absorption into microfluidic channels passivated with and without surface modification (Figure 1). We first performed a series of surface characterization techniques to confirm that the PDMS surface was successfully modified. The LC-MS/MS technique was then utilized to quantify and compare the CBD absorption on the unmodified microchannels and the Teflon-modified microchannels. With the main objective of applying this surface modification to OOC systems, we then further validated that these surface-modified microchannels can support mammalian cell growth as a “precursor” for more complex OOC testing.

### 3.1. Surface Characterizations Confirm the Coating of Teflon AF 2400 on PDMS Surface

**Figure 2:**
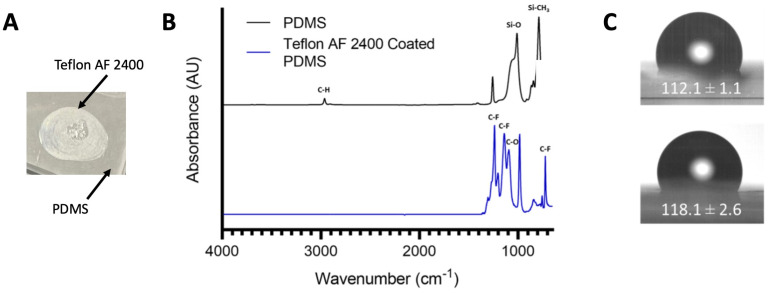
Surface characterizations of PDMS functionalized with and without Teflon AF 2400. (**A**) A photograph of a drop (20 µL) of 1% Teflon AF 2400 incubated on a PDMS for 30 min at 80 °C. A visible spot (~5 mm) could be observed. (**B**) ATR-FTIR spectroscopy of bare PDMS and PDMS coated with Teflon AF 2400. (**C**) Representative images of the water contact angle of bare PDMS (top) and PDMS coated with Teflon AF 2400 (bottom).

We first used flat PDMS surfaces to confirm the fluoropolymer surface functionalization. After a 30 min incubation of 1% Teflon AF 2400 solution in an oven at 80 °C on flat PDMS pieces, we could visually observe a thin layer of the coating forming on top of the PDMS surface as the area coated with Teflon AF 2400 became slightly cloudy (Figure 2A). In ATR-FTIR, PDMS can be recognized by its characteristic absorption bands, such as the C-H stretch at 2960 cm^−1^, the CH_3_ symmetric bending of Si-CH_3_ at 1252 cm^−1^, the Si-O bands at 1063 and 999 cm^−1^, and a peak at 780 cm^−1^ that belongs to the CH_3_ rocking in Si-CH_3_. After coating, new peaks that are attributable to Teflon AF 2400 are clearly present, such as the C-F asymmetric stretch at 1124 cm^−1^ and the C-F symmetric stretch at 1074 cm^−1^ (Figure 2B). This was further confirmed via an XPS analysis, where 55.1 atomic% F was measured after the coating application, while no fluoride was detected in the PDMS (Table 1). The water contact angle measurements were also performed on the PDMS with and without surface modifications (Figure 2C). Typically, a surface with a water contact angle smaller than 90° is considered as a hydrophilic surface, while a surface with a water contact angle greater than 90° is regarded as a hydrophobic surface [37]. Our measurement shows that the unmodified PDMS surface had a contact angle of 112.1° ± 1.1° (Figure 2C). This measurement is consistent with a previous report for PDMS with a water contact angle of 108° ± 7° [18]. After the incubation of the PDMS surface with 1% Teflon AF 2400, the water contact angle slightly increased to 118.1° ± 2.6° (Figure 2C). This further confirms that the PDMS surface was successfully modified.

### 3.2. Azo Coupling Derivatization Method Increased the Limit of Quantification of CBD

**Figure 3:**
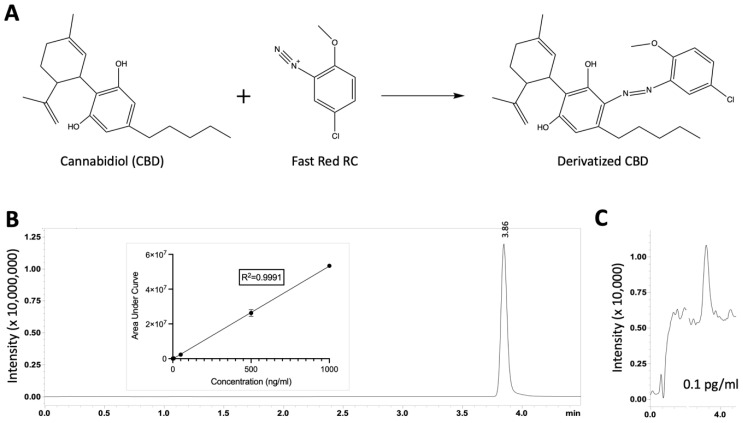
(**A**) Azo coupling between CBD and Fast Red RC, forming derivatized CBD. Derivatization step was performed post-collection. (**B**) A representative ion chromatogram of derivatized CBD (1 µg/mL) resolved at 3.86 min. Insert shows a linear fit calibration curve. (**C**) The LOQ was achieved at 0.1 pg/mL using the derivatization method.

As CBD is a highly hydrophobic and poor ionizing compound, the CBD recovered from the microfluidic channels would fall far below the limit of detection when using the standard LC-MS approach. The derivatization method can significantly increase the ionization efficiency of aromatic compounds [36] and can therefore enhance the sensitivity for detecting CBD from small volumes recovered from microchannels. The first method of azo-coupling-based derivatization for THC and other aromatic compounds was reported by Luo and colleagues [36]. The THC derivatization assay was validated, and the limit of quantification (LOQ) of 0.50 pg/mL was achieved [36]. In this study, we also utilized the azo coupling method to derivatize the CBD to enhance the CBD signal. In fact, CBD and THC have very similar structures [38]. Likewise, the derivatization of CBD with Fast Red RC was also carried out at room temperature (Figure 3A). A calibration curve for the derivatized CBD was generated, and it shows a highly linear correlation (Figure 3B). Furthermore, the lowest CBD concentration (LOQ) that we were able to detect was 0.1 pg/mL for an injection of 2 µL owing to the derivatization method (Figure 3C).

### 3.3. Flat PDMS Surface Functionalized via Teflon AF 2400 Reduced CBD Absorption

**Figure 4:**
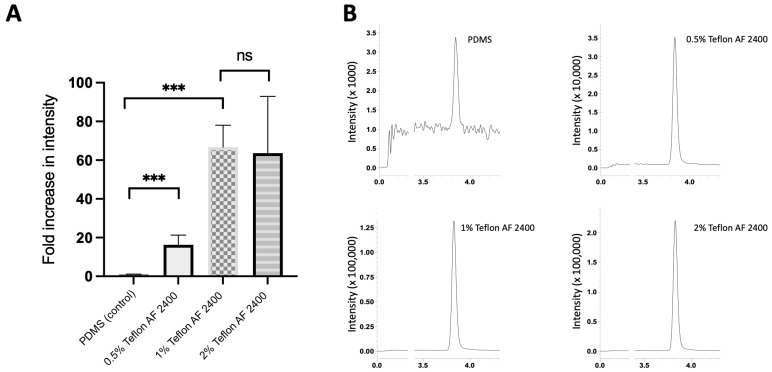
CBD absorption into flat PDMS treated with different concentrations of Teflon AF 2400. (**A**) Comparison of CBD signals after incubation of CBD at 1 µg/mL for 3 h and analyzed via LC-MS/MS. ns, not significantly different (*p* > 0.05); ***, *p* < 0.001 (**B**) Representative ion chromatograms of each surface coating of Teflon AF 2400 (0%, 0.5%, 1%, and 2%).

We first examined the extent of CBD absorption on flat PDMS surfaces modified with different concentrations of Teflon AF 2400, i.e., ranging from 0% to 2%. To this end, 10 µL of CBD at 1 µg/mL was statically incubated on the PDMS surface coated with Teflon AF 2400 and on the control bare PDMS surface. The CBD solution was then collected after the experiment, and the derivatization process was performed. For a bare PDMS without surface modification, there was a significant loss of CBD via absorption into PDMS (Figure 4A), and only a weak peak was detected using LC-MS/MS (Figure 4B). On the other hand, we identified an increase of approximately 20 times in the signal intensity when the PDMS surface was modified with 0.5% Teflon AF 2400 (Figure 4). When the fluoropolymer concentration was increased to 1%, the CBD detection sensitivity increased by 60 times (Figure 4). However, when the fluoropolymer concentration was increased to 2%, no further increase in the CBD signal was observed, indicating that the Teflon AF 2400 coating onto the PDMS surface might have reached saturation (Figure 4). In fact, a range of CBD concentrations from 0.1 μg/mL to 10 μg/mL on selected surfaces were also tested (data not shown). However, for testing with the high concentration of CBD (10 μg/mL), the output signal from LC-MS/MS reached saturation, which would make further analysis inaccurate. On the other hand, at the lower concentration of CBD (0.1 μg/mL), most of the CBD was absorbed when incubated on the unmodified PDMS, the output LC-MS/MS signal was mostly reduced to background signal, and it was difficult to make further comparisons to other surface modifications. Hence, we chose 1 μg/mL as the most optimal model concentration for testing the extent of CBD absorption in different surface modifications in all following experiments.

### 3.4. Glass Syringe and PEEK Tubing Combination Increased the CBD Recovery in Flow Conditions

**Figure 5:**
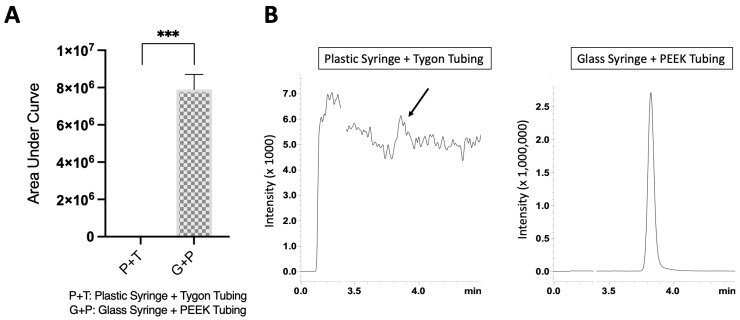
CBD recovery comparison from different delivery setups. (**A**) Plastic polypropylene syringe and Tygon tubing delivery setup had significant loss of CBD in comparison to glass syringe/PEEK tubing setup. ***, *p* < 0.001. (**B**) Representative ion chromatogram for each delivery setup. (N = 3).

It is a common approach in the microfluidic research area to use a pump device (e.g., syringe pump, peristaltic pump, pneumatic pump, or pressure-driven pump) to deliver fluids from an external reservoir into the microfluidic chips [39]. Syringe pumps are probably a more widely used pumping system due to their ease of setup and being cheaper than other pump systems. In a typical syringe-pump-driven experiment, a syringe is usually loaded with fluids of interest (e.g., test drugs) and connected to a microfluidic chip via connecting tubings. Single-use plastic syringes made from polypropylene and plastic Tygon tubings are also very common choices [39]. However, it was also reported that some laboratory plastic materials can inadvertently absorb certain testing compounds [40]. As CBD is a very hydrophobic compound, we wanted to verify if the common fluid delivery setup (i.e., polypropylene syringe and plastic Tygon tubing) was suitable for such a purpose. We quantified the extent of CBD loss when it was loaded into a disposable polypropylene plastic syringe and connected with plastic Tygon tubing after perfusion for 3 h. Surprisingly, we observed that the CBD was almost completely lost by the plastic polypropylene syringe/Tygon tubing delivery system (Figure 5). We then conducted the exact same experiments using glass syringes and polyetheretherketone (PEEK) tubings for connecting the glass syringe to microfluidic chips. In fact, PEEK tubings are thermoplastic polymers that are chemically inert to most solvents and have become a standard in the operation of many HPLC systems. In comparison to the delivery system using disposable plastic polypropylene syringe/Tygon tubing, we observed that when using a glass syringe and PEEK tubing combination, the sensitivity of the CBD signal from the collected eluent dramatically increased (Figure 5), and we observed a sharp and strong peak (glass syringe + PEEK tubing) in comparison to a near background noise peak with the polypropylene plastic syringe and Tygon tubing (Figure 5B). Hence, for all the following experiments, the glass syringe and PEEK tubing combination was used for the delivery of CBD in microfluidic channels.

### 3.5. Microfluidic Channels Functionalized via Teflon AF 2400 Improved CBD Recovery

**Figure 6:**
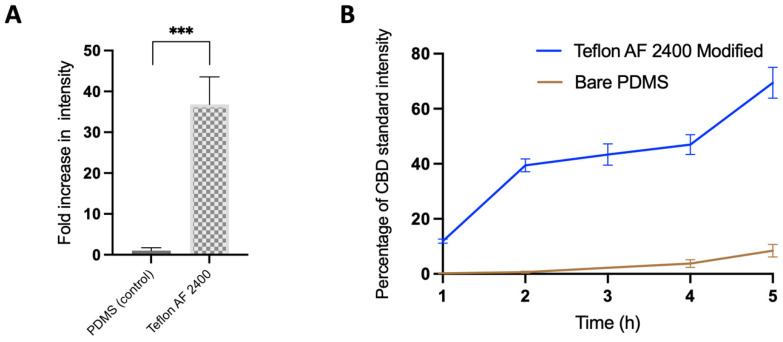
Quantification of CBD absorption into untreated microchannels and microchannels treated with 1% Teflon AF 2400. (**A**) CBD at 1 µg/mL was statically incubated inside a microchannel for 3 h or (**B**) was perfused through the microchannels using a syringe pump for 5 h. ***, *p* < 0.001. The eluent concentrations were derivatized and quantified via LC-MS/MS at the end of each hour (N = 3).

We then quantified the CBD absorption in microfluidic channels with surface modification using Teflon AF 2400. We first compared the unmodified microchannels and the microchannels modified with 1% Teflon AF 2400 under static incubations of CBD, as some OOC systems are more suitable for cell culture under static conditions [41]. Interestingly, we were able to detect a 35-fold increase in the CBD signal from the surface-modified microchannels compared to the bare PDMS channels (Figure 6A). A similar trend was also previously observed for the flat surface PDMS modified with 1% Teflon AF 2400 (Figure 4A).

Furthermore, we compared the CBD recovery rates of the PDMS microfluidic channels modified with Teflon AF 2400 and the unmodified PDMS microfluidic channels under the flow condition (at 2 µL/min) using the glass syringe/PEEK tubing combination as the delivery setup. As can be seen from Figure 6B, in the first hour of CBD perfusion, the microchannel treated with Teflon AF 2400 had an increase of over ~10x in sensitivity compared to the control bare PDMS microchannel. As expected, the longer the perfusion time of CBD, the higher the detected CBD signal was from the microchannel exit (Figure 6B). In fact, after 5 h of perfusion, the CBD signal from the surface-modified channel reached ~70% of the CBD standard and was ~15 times higher than the signal from the microchannels without the surface modification (Figure 6B).

### 3.6. Microfluidic Surface Functionalized with Teflon AF 2400 Is Suitable for Cell Experiment

**Figure 7:**
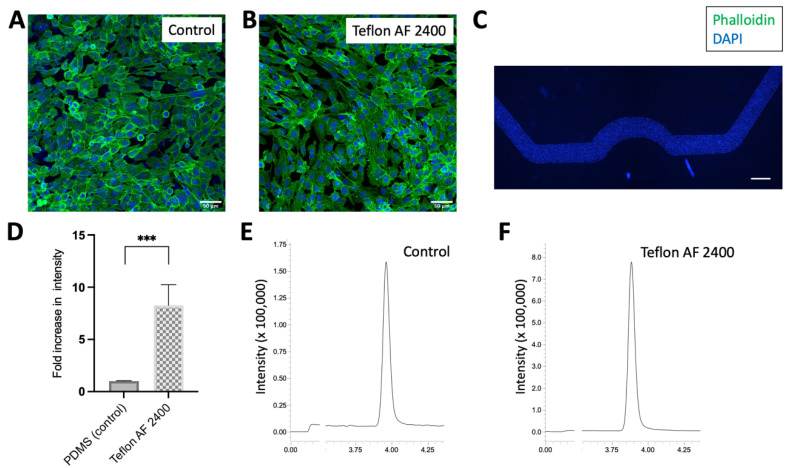
Morphological assessment and testing of CBD compound on hCMEC/D3 cell-cultured microchannels. D3 cells were cultured on microchannels (**A**) without and (**B**) with 1% Teflon AF 2400 surface modification. Phalloidin (Green) and DAPI (Blue) stained cell morphology after 2 days in culture. (**C**) Low magnification showing the entire channel of uniformly distributed cell culture (DAPI stained cell) in channel coated with 1% Teflon AF 2400. Scale bars: (**A**,**B**), 50 µm; (**C**), 500 µm. (**D**) CBD at 1 µg/mL was perfused over unmodified (control) microchannels and microchannels modified with 1% Teflon AF 2400 cultured with hCMEC/D3 cells for 3 h. The eluents were collected and quantified using LC-MS/MS. ***, *p* < 0.001. Representative of ion chromatograms of control (**E**) and microchannels coated with 1% Teflon AF 2400 (**F**) cultured with hCMEC/D3 cells (N = 3).

We have thus far demonstrated that the PDMS surfaces functionalized with Teflon AF 2400 (in both the flat PDMS surface and inside the PDMS microchannels) have superior performance than the bare PDMS surfaces in reducing the hydrophobic compound (i.e., CBD) absorptions. This is a critical step for extending this technique to OOC systems, especially for analytical drug discovery applications. The next step we took was to ensure that the microfluidic channels modified with Teflon AF 2400 were suitable for cell culture, and to ensure that such modified surface could still support normal cell growth and function. This step is considered as a “precursor” towards functional OOC systems. As a model cell line, we utilized hCMEC/D3 cells to test the suitability of such functionalized PDMS channels for the culture of mammalian cells on a chip. hCMEC/D3 cells were cultured inside the microfluidic chips with and without the Teflon AF 2400 coating for over 2 days. Similar cell morphologies were observed after the culture period via staining with Phalloidin and DAPI (Figure 7A,B). This demonstrates that the surface coated with Teflon AF 2400 can support normal mammalian cell growth. By scanning the full length of the microfluidic channel, we observed that the hCMEC/D3 cells were uniformly distributed across the entire microchannel modified with Teflon AF 2400 (Figure 7C). Finally, CBD was perfused over the microchannels cultured with hCMEC/D3 cells for 3 h, and the surface-modified microchannel had a CBD sensitivity that was nearly eight-fold higher than the bare PDMS microchannels (Figure 7D–F). In fact, culturing hCMEC/D3 cells on a chip, especially as a tight monolayer, is a common model for blood–brain barrier (BBB) research [42]. Those BBB on-chip models are also becoming popular in vitro models to be explored in neurological diseases and brain cancer studies [12]. Although it was reported that cannabinoids, including CBD, can be beneficial in treating patients with brain cancers, there is still more work required to thoroughly investigate the use of CBD as an anticancer agent [26]. We believe that the in vitro OOC system approach, especially with an improved surface modification to ensure there is no loss of quantifying drug compounds, can be beneficial to further understand the CBD mechanism of action as an anticancer agent.

## 4. Conclusions

This paper reports a simple one-step methodology to modify a PDMS surface, using a material that is very popular in OOC platforms, especially for drug discovery. Here, we propose the use of OOC systems for testing cannabinoid compounds, particularly CBD, against different disease types due to the rising popularity of exploring cannabinoids for their medicinal values [26]. The critical analysis of CBD absorption in OOC platforms and a method to reduce the absorption should bridge the gap for OOC’s wider applications in CBD testing.

We demonstrated the passivation of a PDMS surface with Teflon AF 2400 solution to be able to form a non-adhesive layer to CBD. We found that 1% of Teflon AF 2400 was an effective concentration to optimally reduce CBD absorption. Furthermore, we also demonstrated the effectiveness of using a glass syringe and PEEK tubing combination as a fluidic pumping setup rather than using a plastic syringe and plastic tubing approach to minimize drug compound loss during fluid transport. Using the microchannels modified with 1% Teflon AF 2400, we were able to culture hCMEC/D3 without observing any morphological differences compared to the bare PDMS microchannels. Furthermore, the sensitivity of the CBD tested from the surface-modified microchannels was much higher (8x) than the unmodified microchannels.

Even though the focus of this paper is to demonstrate that the PDMS surface modified with Teflon AF 2400 can significantly reduce the CBD absorption, in order to successfully implement this technique to broader OOC applications, some important factors still need to be addressed. Firstly, we would need to ensure that the Teflon coating can remain stable, both chemically and mechanically, for the duration of the cell culture experiments. For typical OOC systems, the duration of the cell culture can range from weeks to months [43]. Secondly, different cell types and their responses should be tested on the Teflon-modified surface. In this paper, we only performed the morphological assessment of the hCMEC/D3 cell line and their permeability, and other basic cellular functions still need to be validated [12]. Lastly, a broad range of different types of pharmaceutical compounds, e.g., compounds with different hydrophobicities and sizes, should also be carefully examined. We believe that such validation steps are critical to ensure that our reported simple one-step surface modification method can be fully adopted and to broaden the impact of using various OOC systems in drug discovery applications.

## Figures and Tables

**Table 1:** The atomic% for PDMS and PDMS coated with Teflon AF 2400 as determined via XPS (N = 3).

	PDMS (Atomic%)	PDMS Coated with Teflon AF 2400 (Atomic%)
F	0.0 ± 0.0	55.1 ± 0.1
O	26.9 ± 0.1	11.7 ± 0.1
C total	48.4 ± 0.1	32.8 ± 0.2
C1	41.2 ± 0.0	0.6 ± 0.2
C2	6.9 ± 0.2	0.3 ± 0.1
C3	0.2 ± 0.0	0.3 ± 0.0
C4	0.1 ± 0.0	0.4 ± 0.0
C5	0.0 ± 0.0	1.5 ± 0.0
C6	0.0 ± 0.0	9.0 ± 0.1
C7	0.0 ± 0.0	8.9 ± 0.0
C8	0.0 ± 0.0	11.7 ± 0.0
Si	24.7 ± 0.2	0.5 ± 0.0

## Data Availability

Available upon request from the corresponding authors.

## References

[1] Koyilot M.C., Natarajan P., Hunt C.R., Sivarajkumar S., Roy R., Joglekar S., Pandita S., Tong C.W., Marakkar S., Subramanian L. (2022). Breakthroughs and Applications of Organ-on-a-Chip Technology. Cells.

[2] Mathur A., Loskill P., Shao K., Huebsch N., Hong S., Marcus S.G., Marks N., Mandegar M., Conklin B.R., Lee L.P. (2015). Human iPSC-based Cardiac Microphysiological System For Drug Screening Applications. Sci. Rep..

[3] Legendre A., Baudoin R., Alberto G., Paullier P., Naudot M., Bricks T., Brocheton J., Jacques S., Cotton J., Leclerc E. (2013). Metabolic characterization of primary rat hepatocytes cultivated in parallel microfluidic biochips. J. Pharm. Sci..

[4] Sutterby E., Thurgood P., Baratchi S., Khoshmanesh K., Pirogova E. (2020). Microfluidic Skin-on-a-Chip Models: Toward Biomimetic Artificial Skin. Small.

[5] Musah S., Dimitrakakis N., Camacho D.M., Church G.M., Ingber D.E. (2018). Directed differentiation of human induced pluripotent stem cells into mature kidney podocytes and establishment of a Glomerulus Chip. Nat. Protoc..

[6] Shrestha J., Razavi Bazaz S., Aboulkheyr Es H., Yaghobian Azari D., Thierry B., Ebrahimi Warkiani M., Ghadiri M. (2020). Lung-on-a-chip: The future of respiratory disease models and pharmacological studies. Crit. Rev. Biotechnol..

[7] Kim H.J., Ingber D.E. (2013). Gut-on-a-Chip microenvironment induces human intestinal cells to undergo villus differentiation. Integr. Biol..

[8] Ragelle H., Goncalves A., Kustermann S., Antonetti D.A., Jayagopal A. (2020). Organ-On-A-Chip Technologies for Advanced Blood-Retinal Barrier Models. J. Ocul. Pharmacol. Ther..

[9] Kimura H., Sakai Y., Fujii T. (2018). Organ/body-on-a-chip based on microfluidic technology for drug discovery. Drug Metab. Pharmacokinet..

[10] Maschmeyer I., Lorenz A.K., Schimek K., Hasenberg T., Ramme A.P., Hübner J., Lindner M., Drewell C., Bauer S., Thomas A. (2015). A four-organ-chip for interconnected long-term co-culture of human intestine, liver, skin and kidney equivalents. Lab Chip.

[11] Sung J.H., Shuler M.L. (2009). A micro cell culture analog (microCCA) with 3-D hydrogel culture of multiple cell lines to assess metabolism-dependent cytotoxicity of anti-cancer drugs. Lab Chip.

[12] Peng B., Hao S., Tong Z., Bai H., Pan S., Lim K.-L., Li L., Voelcker N.H., Huang W. (2022). Blood–brain barrier (BBB)-on-a-chip: A promising breakthrough in brain disease research. Lab Chip.

[13] Cherusseri J., Savio C.M., Khalid M., Chaudhary V., Numan A., Varma S.J., Menon A., Kaushik A. (2022). SARS-CoV-2-on-Chip for Long COVID Management. Biosensors.

[14] Zhang B., Radisic M. (2017). Organ-on-a-chip devices advance to market. Lab Chip.

[15] Campbell S.B., Wu Q., Yazbeck J., Liu C., Okhovatian S., Radisic M. (2021). Beyond Polydimethylsiloxane: Alternative Materials for Fabrication of Organ-on-a-Chip Devices and Microphysiological Systems. ACS Biomater. Sci. Eng..

[16] Cameron T.C., Randhawa A., Grist S.M., Bennet T., Hua J., Alde L.G., Caffrey T.M., Wellington C.L., Cheung K.C. (2022). PDMS Organ-On-Chip Design and Fabrication: Strategies for Improving Fluidic Integration and Chip Robustness of Rapidly Prototyped Microfluidic In Vitro Models. Micromachines.

[17] Regehr K.J., Domenech M., Koepsel J.T., Carver K.C., Ellison-Zelski S.J., Murphy W.L., Schuler L.A., Alarid E.T., Beebe D.J. (2009). Biological implications of polydimethylsiloxane-based microfluidic cell culture. Lab Chip.

[18] Gokaltun A., Yarmush M.L., Asatekin A., Usta O.B. (2017). Recent advances in nonbiofouling PDMS surface modification strategies applicable to microfluidic technology. Technology.

[19] Khetani S., Yong K.W., Ozhukil Kollath V., Eastick E., Azarmanesh M., Karan K., Sen A., Sanati-Nezhad A. (2020). Engineering Shelf-Stable Coating for Microfluidic Organ-on-a-Chip Using Bioinspired Catecholamine Polymers. ACS Appl. Mater. Interfaces.

[20] Tu Q., Wang J.-C., Zhang Y., Liu R., Liu W., Ren L., Shen S., Xu J., Zhao L., Wang J. (2012). Surface modification of poly(dimethylsiloxane) and its applications in microfluidics-based biological analysis. Rev. Anal. Chem..

[21] Tan S.H., Nguyen N.T., Chua Y.C., Kang T.G. (2010). Oxygen plasma treatment for reducing hydrophobicity of a sealed polydimethylsiloxane microchannel. Biomicrofluidics.

[22] Hellmich W., Regtmeier J., Duong T.T., Ros R., Anselmetti D., Ros A. (2005). Poly(oxyethylene) Based Surface Coatings for Poly(dimethylsiloxane) Microchannels. Langmuir.

[23] Chuah Y.J., Kuddannaya S., Lee M.H., Zhang Y., Kang Y. (2015). The effects of poly(dimethylsiloxane) surface silanization on the mesenchymal stem cell fate. Biomater. Sci..

[24] Radisic M., Loskill P. (2021). Beyond PDMS and Membranes: New Materials for Organ-on-a-Chip Devices. ACS Biomater. Sci. Eng..

[25] Ren K., Dai W., Zhou J., Su J., Wu H. (2011). Whole-Teflon microfluidic chips. Proc. Natl. Acad. Sci. USA.

[26] Doherty G.J., de Paula B.H.R. (2021). Cannabinoids in glioblastoma multiforme-hype or hope?. Br. J. Cancer.

[27] Zhelyazkova M., Kirilov B., Momekov G. (2020). The pharmacological basis for application of cannabidiol in cancer chemotherapy. Pharmacia.

[28] Jhawar N., Schoenberg E., Wang J.V., Saedi N. (2019). The growing trend of cannabidiol in skincare products. Clin. Dermatol..

[29] Gupta A.K., Talukder M. (2021). Cannabinoids for skin diseases and hair regrowth. J. Cosmet. Dermatol..

[30] Navarrete F., Gasparyan A., Manzanares J. (2022). CBD-mediated regulation of heroin withdrawal-induced behavioural and molecular changes in mice. Addict. Biol..

[31] Zieba J., Sinclair D., Sebree T., Bonn-Miller M., Gutterman D., Siegel S., Karl T. (2019). Cannabidiol (CBD) reduces anxiety-related behavior in mice via an FMRP-independent mechanism. Pharmacol. Biochem. Behav..

[32] Verrico C.D., Wesson S., Konduri V., Hofferek C.J., Vazquez-Perez J., Blair E., Dunner K., Salimpour P., Decker W.K., Halpert M.M. (2020). A randomized, double-blind, placebo-controlled study of daily cannabidiol for the treatment of canine osteoarthritis pain. Pain.

[33] Alhamoruni A., Lee A.C., Wright K.L., Larvin M., O’Sullivan S.E. (2010). Pharmacological effects of cannabinoids on the Caco-2 cell culture model of intestinal permeability. J. Pharmacol. Exp. Ther..

[34] Mangal N., Erridge S., Habib N., Sadanandam A., Reebye V., Sodergren M.H. (2021). Cannabinoids in the landscape of cancer. J. Cancer Res. Clin. Oncol..

[35] Delon L.C., Nilghaz A., Cheah E., Prestidge C., Thierry B. (2020). Unlocking the Potential of Organ-on-Chip Models through Pumpless and Tubeless Microfluidics. Adv. Healthc. Mater..

[36] ul Ahmad A., Liang H., Ali S., Abbas Q., Farid A., Ali A., Iqbal M., Ahmad khan I., Pan L., Abbas A. (2020). Cheap, reliable, reusable, thermally and chemically stable fluorinated hexagonal boron nitride nanosheets coated Au nanoparticles substrate for surface enhanced Raman spectroscopy. Sens. Actuators B Chem..

[37] Akther F., Yakob S.B., Nguyen N.T., Ta H.T. (2020). Surface Modification Techniques for Endothelial Cell Seeding in PDMS Microfluidic Devices. Biosensors.

[38] Atakan Z. (2012). Cannabis, a complex plant: Different compounds and different effects on individuals. Ther. Adv. Psychopharmacol..

[39] Kaarj K., Yoon J.Y. (2019). Methods of Delivering Mechanical Stimuli to Organ-on-a-Chip. Micromachines.

[40] Malysheva A., Ivask A., Hager C., Brunetti G., Marzouk E.R., Lombi E., Voelcker N.H. (2016). Sorption of silver nanoparticles to laboratory plastic during (eco)toxicological testing. Nanotoxicology.

[41] Fedi A., Vitale C., Fato M., Scaglione S. (2023). A Human Ovarian Tumor & Liver Organ-on-Chip for Simultaneous and More Predictive Toxo-Efficacy Assays. Bioengineering.

[42] Brown T.D., Nowak M., Bayles A.V., Prabhakarpandian B., Karande P., Lahann J., Helgeson M.E., Mitragotri S. (2019). A microfluidic model of human brain (μHuB) for assessment of blood brain barrier. Bioeng. Transl. Med..

[43] Hsieh H.L., Nath P., Huang J.H. (2019). Multistep Fluidic Control Network toward the Automated Generation of Organ-on-a-Chip. ACS Biomater. Sci. Eng..

